# p53- and ERK7-Dependent Ribosome Surveillance Response Regulates *Drosophila* Insulin-Like Peptide Secretion

**DOI:** 10.1371/journal.pgen.1004764

**Published:** 2014-11-13

**Authors:** Kiran Hasygar, Ville Hietakangas

**Affiliations:** Department of Biosciences & Institute of Biotechnology, University of Helsinki, Helsinki, Finland; University of California San Francisco, United States of America

## Abstract

Insulin-like signalling is a conserved mechanism that coordinates animal growth and metabolism with nutrient status. In *Drosophila*, insulin-producing median neurosecretory cells (IPCs) regulate larval growth by secreting insulin-like peptides (dILPs) in a diet-dependent manner. Previous studies have shown that nutrition affects dILP secretion through humoral signals derived from the fat body. Here we uncover a novel mechanism that operates cell autonomously in the IPCs to regulate dILP secretion. We observed that impairment of ribosome biogenesis specifically in the IPCs strongly inhibits dILP secretion, which consequently leads to reduced body size and a delay in larval development. This response is dependent on p53, a known surveillance factor for ribosome biogenesis. A downstream effector of this growth inhibitory response is an atypical MAP kinase ERK7 (ERK8/MAPK15), which is upregulated in the IPCs following impaired ribosome biogenesis as well as starvation. We show that ERK7 is sufficient and essential to inhibit dILP secretion upon impaired ribosome biogenesis, and it acts epistatically to p53. Moreover, we provide evidence that p53 and ERK7 contribute to the inhibition of dILP secretion upon starvation. Thus, we conclude that a cell autonomous ribosome surveillance response, which leads to upregulation of ERK7, inhibits dILP secretion to impede tissue growth under limiting dietary conditions.

## Author Summary

Ribosome biogenesis is a major consumer of cellular energy and a rate-limiting process during cell growth. The ribosome biogenesis pathway is tightly connected with signaling pathways that regulate tissue growth. For example, nutrient-regulated signaling cues adjust the rate of ribosome biogenesis. On the other hand, the process of ribosome biogenesis is closely monitored by so-called surveillance mechanisms. The best-known ribosome surveillance factor is the transcription factor and tumor suppressor p53. In proliferating cells, activation of p53 upon disturbed ribosome biogenesis leads to cell cycle arrest and inhibition of proliferation. Here we show that ribosome surveillance not only regulates growth locally in proliferating cells, but is also coupled to hormonal growth control through regulation of insulin like peptide (dILPs) secretion. We observed that inhibition of ribosome biogenesis in the *Drosophila* insulin-producing cells generates a strong cell autonomous signal to inhibit dILP secretion. We identify two downstream effectors of this ribosome surveillance response by showing that p53 as well as an atypical MAP kinase ERK7 are mediators of the inhibition of dILP secretion. We also provide evidence that this ribosome surveillance mechanism contributes to nutrient-dependent regulation of dILP secretion.

## Introduction

Animals coordinate tissue growth and body size with changing nutrient conditions and it is well established that insulin-like signalling has a key role in this process [1], [2]. In mammals, insulin-like signalling is mediated by insulin and insulin-like growth factors through their respective receptors. *Drosophila* possesses a single insulin-like receptor, which is activated by insulin-like peptides (dILPs). There are currently eight dILPs identified in *Drosophila* and several of them (e.g. dILP2, -3 and -5) are mainly expressed and secreted by a group of 14 median neurosecretory cells, also known as insulin-producing cells (IPCs) [3], [4]. These cells are critical in controlling organismal growth, as disturbance of IPC function leads to strongly reduced body size [4]. IPC function is coupled to nutrient status as starvation of *Drosophila* larvae leads to inhibition of dILP secretion [5]. Recent studies have demonstrated that fat body, the insect counterpart of adipose tissue and liver, senses nutrient status and regulates dILP secretion by hormonal mechanisms [5], [6]. Secreted fat body cytokine Unpaired 2 is regulated by dietary sugars and lipids and it controls a population of GABAergic neurons in the brain that project onto IPCs. Upd2 activates the JAK-STAT pathway in these neurons, which relieves the inhibitory effect of GABAergic neurons on IPCs, resulting in dILP secretion [6]. Moreover, the fat body senses amino acid levels and in turn secretes an unknown humoral signal, which facilitates dILP secretion from the IPCs [5]. Recently, it was shown that adiponectin receptor also regulates dILP secretion from the IPCs, but the corresponding ligand is not yet identified [7]. Beyond these examples, the regulation of IPC function in response to changing nutrient status is poorly understood. In particular, very little is known of how cell intrinsic pathways within the IPCs affect dILP secretion, and consequently organismal growth.

Ribosome biogenesis is a major consumer of cellular energy and a key determinant of cell growth capacity [8]. Ribosome biogenesis is tightly regulated by nutrient, growth factor and stress responsive signalling networks [1], [8]–[13]. As the ribosome biogenesis pathway is a major integrator of growth regulatory signals, its activity needs to be closely monitored. p53 serves as one of the ribosome biogenesis surveillance factors [14]. Impaired ribosome biogenesis activates p53, likely through several parallel mechanisms. For example, imbalance of ribosomal components allows ribosomal proteins RPL5 and RPL11 in complex with 5S rRNA to inhibit HDM2/MDM2-dependent degradation of p53 [15]–[17]. On the other hand, nucleolar Myb binding protein 1A (MYBBP1A) translocates to the nucleoplasm if rRNA production is inhibited [18]. Nucleoplasmic MYBBP1A increases p53 activity by promoting its tetramerization and acetylation [19]. In proliferating cells, activation of p53 through these mechanisms leads to cell cycle arrest [16]. However, the role of ribosome biogenesis surveillance in other physiological settings is poorly understood. This is a relevant question, as disturbed ribosome biogenesis is known to have highly tissue-specific consequences *in vivo*. In humans, mutations in ribosomal components or other ribosome biogenesis genes lead to genetic disorders called ribosomopathies [20]. They manifest, for example, as bone marrow failure, anemia, skin abnormalities, and pancreatic insufficiency [20]. In *Drosophila*, mutations that disturb ribosome biogenesis lead to so-called *Minute* phenotype, displaying impaired growth, poor fertility and short and thin bristles [21]. Inhibition of ribosome biogenesis specifically in the *Drosophila* fat body or prothoracic gland influences systemic growth by affecting hormonal regulation through insulin and ecdysone signalling axes, respectively [22]–[24]. Which signalling mechanisms mediate the distinct cell type-specific phenotypes of ribosome surveillance *in vivo* is, however, poorly understood.

Here we report that dILP secretion is tightly coupled to the activity of the ribosome biogenesis pathway within the IPCs. We show that the p53 dependent ribosomal surveillance response inhibits dILP secretion and identify atypical MAP kinase ERK7 as a downstream effector of the ribosome surveillance response in this setting. Furthermore, we provide evidence that both p53 and ERK7 activities within the IPCs contribute to regulation of dILP secretion in response to nutrient status. Thus, we propose that the p53- and ERK7-dependent ribosome surveillance pathway serves as a local branch of the IPC-regulating nutrient sensing network, parallel to the humoral signals derived from the fat body.

## Results

### Kinome-wide screen identifies novel regulators of IPC function

**Figure 1:**
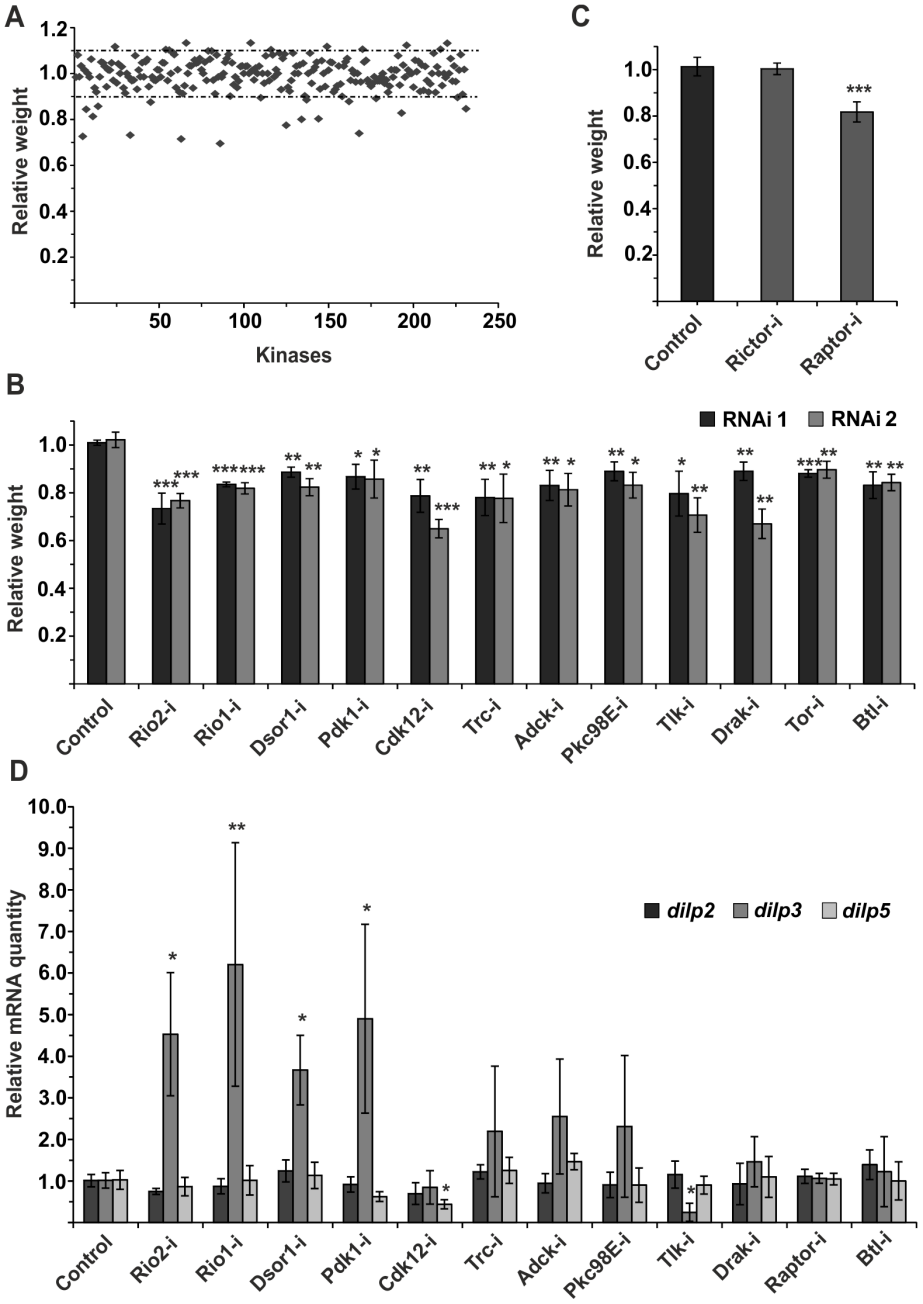
Kinome-wide screen identifies novel regulators of IPCs. (**A**) Overview of the kinome-wide RNAi screen in the IPCs. Each point indicates the mean body weight of RNAi expressing flies normalized to weight of flies from the same vial that do not express RNAi in the IPCs (i.e. relative weight). Dotted lines indicate +/−10% of the median body weight. (**B**) Kinome-wide RNAi screen identifies 12 kinases whose knockdown in the IPCs by two independent RNAi lines leads to significantly reduced body weight. Error bars represent standard deviation (N≥3, ≥10 flies/group). (**C**) TORC1 is essential for normal IPC function. Knockdown of Raptor, but not Rictor, in IPCs leads to reduction in body weight. Error bars represent standard deviation (N = 4, ≥10 flies/group). (**D**) Relative mRNA expression of *dilp2*, *dilp3*, and *dilp5* upon knockdown of the kinase hits. Knockdown of Raptor, instead of TOR, was used to inhibit TORC1 function. Error bars represent standard deviation (N = 3, 10 brains/group). GAPDH was used as an internal reference. *p<0.05, **p<0.01, ***p<0.001 (Student's t-test).

While the role of *Drosophila* insulin-like peptides (dILPs) in systemic growth control is well established, it remains poorly understood how the secretion of dILPs is regulated in response to changing dietary conditions. To get a better insight into this question, we explored the cell autonomous signalling mechanisms that regulate dILP secretion, by performing a kinome-wide screen with RNAi expressed specifically in the IPCs. Impaired IPC function compromises tissue growth [4] and therefore we used body weight of emerging adults as a screening readout (Figure 1A, Table S1). To normalize variation between vials due to growth conditions, we determined the relative weight wherein the mean weight of the flies expressing both dILP2-Gal4 and RNAi was divided by the mean weight of control flies (not expressing the dILP2-Gal4) from the same vial. In total, 231 kinases were screened. All primary hits displaying >10% difference to the median relative weight were further analyzed by an independent RNAi line. Altogether 12 protein kinases showed significant body weight reduction in two independent RNAi lines (Figure 1B). All identified kinases have mammalian orthologs (Table 1). In addition to these 12 kinases identified here, we have earlier shown that atypical PKC is essential for IPC function [25]. One of the kinase hits was TOR (target of rapamycin). TOR kinase is present in two functionally distinct complexes, TOR complex 1 and 2 (TORC1 and TORC2). To explore which of the TOR complexes have a regulatory role in the IPCs, we depleted Raptor and Rictor, essential components of TORC1 and TORC2, respectively. Knockdown of Raptor in the IPCs significantly reduced total body weight, while depletion of Rictor had no significant impact (Figure 1C), showing that TORC1 regulates growth through the IPCs. However, as RNAi does not completely silence gene expression, we cannot rule out the involvement of TORC2.

**Table 1:** Brief descriptions of the confirmed hits and their mammalian homologs.

Gene	CG number	Mammalian homolog	Brief description
Rio2	CG11859	RIO2/RIOK2	Rio kinase 2; required for processing of 18S rRNA and maturation of 40S ribosomal subunit.
Rio1	CG11660	RIO1/RIOK1	Rio kinase 1; required for processing of 18S rRNA and maturation of 40S ribosomal subunit.
Dsor1/d-MEK	CG15793	MAP2K1	Mitogen-activated protein kinase kinase 1; activates ERK1/2 MAP kinases.
Pdk1	CG1210	PDPK1	3-phosphoinositide dependent protein kinase-1; phosphorylates the activation loop of many AGC kinases, including AKT, S6K, and PKC.
Cdk12	CG7597	CDK12	Cyclin-dependent kinase 12; transcription elongation-associated CTD kinase. Involved in cell cycle, maintenance of genome stability and in RNA splicing.
Trc	CG8637	STK38	Serine/threonine kinase 38; controls cell structure and proliferation of a variety of polarized outgrowths.
Adck	CG3608	ADCK1	aarF domain containing kinase 1; mitochondrial protein, might be involved in coenzyme Q (10) biosynthesis.
Pkc98E	CG1954	PKCE	Protein kinase C, epsilon; calcium-independent, phospholipid and diacylglycerol-dependent, serine- and threonine-specific kinase with many functions.
Tlk	CG34412	TLK1	Tousled-like kinase 1; regulates chromatin dynamics, DNA replication and repair, transcription, chromosome segregation.
Drak	CG32666	DRAK1/STK17A	Serine/threonine kinase 17a; regulates cytoskeletal dynamics and morphogenesis.
Tor	CG5092	mTOR	Mechanistic target of rapamycin (serine/threonine kinase); kinase subunit of both mTORC1 and mTORC2, regulates cell growth and metabolism in response to nutrient and hormonal signals.
Btl	CG32134	FGFR2	Fibroblast growth factor receptor 2; regulates many developmental processes, including branching morphogenesis.

To explore gene expression of three IPC-expressed *dilp* genes, namely *dilp2*, *dilp3* and *dilp5*, we analyzed RNA from larval brains by quantitative RT-PCR (Figure 1D). In the case of most hits, expression of *dilp2* and *dilp5* remained nearly unchanged. In contrast, *dilp3* expression was elevated in several samples. This is likely due to a feedback mechanism activated by reduced autocrine insulin signalling in the IPCs [26]. Only depletion of Tousled-like kinase (Tlk) significantly reduced *dilp3* expression (Figure 1D).

### Disturbed ribosome biogenesis in the IPCs inhibits dILP secretion

**Figure 2:**
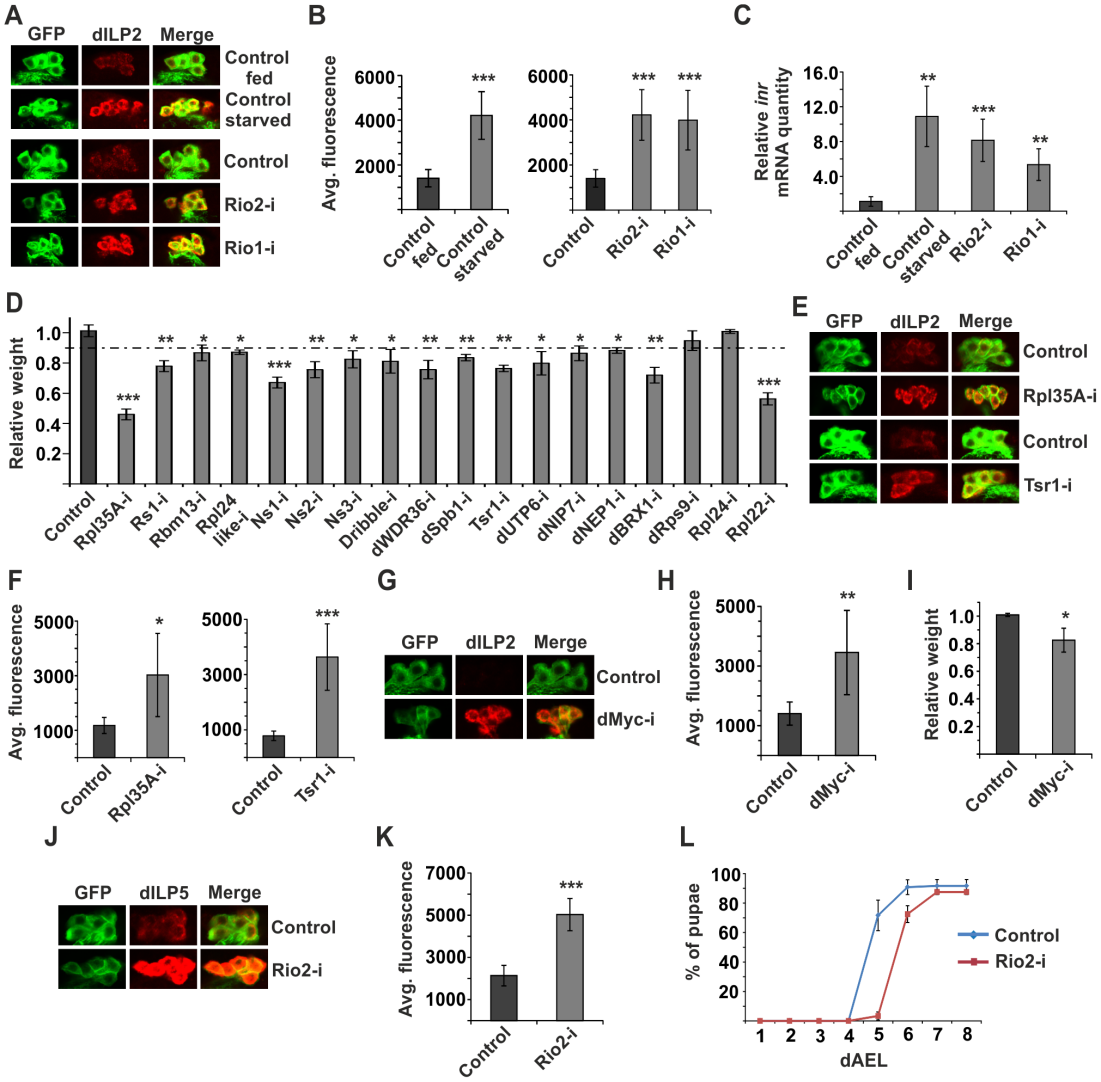
Disturbed ribosome biogenesis in the IPCs inhibits dILP secretion. (**A**) Starvation or disturbed ribosome biogenesis by knockdown of Rio1 or Rio2 leads to accumulation of dILP2 in cell bodies of IPCs. (**B**) Quantification of the immunofluorescence from Fig. 2A. Error bars represent standard deviation (N≥10) (**C**) Relative mRNA expression of *inr* in whole larvae is elevated upon starvation or knockdown of Rio1 or Rio2 in the IPCs. Error bars represent standard deviation (N = 3, ≥5 larvae/group). *cdk7* was used as an internal reference. (**D**) IPC-specific knockdown of ribosomal components or genes involved in ribosome biogenesis leads to reduction of body weight. Error bars represent standard deviation (N≥3, ≥10 flies/group). (**E, F**) Knockdown of Rpl35A or Tsr1 inhibits dILP2 secretion from IPCs. Error bars represent standard deviation (N≥10). (**G, H**) Depletion of dMyc in the IPCs leads to accumulation of dILP2. Error bars represent standard deviation (N≥10). (**I**) dMyc knockdown in the IPCs leads to reduced body weight. Error bars represent standard deviation (N≥3, ≥10 flies/group). (**J, K**) Depletion of Rio2 in the IPCs leads to accumulation of dILP5. Error bars represent standard deviation (N≥10). (**L**) Rio2 knockdown in the IPCs leads to delay in pupation. Error bars represent standard deviation (N = 4, 30 larvae/group). For all confocal images, IPCs are labelled by GFP (green) and dILP2 or dILP5 is shown as red. *p<0.05, **p<0.01, ***p<0.001 (Student's t-test).

Secretion of dILPs is a key regulatory level in determining the activity of systemic insulin signalling [5]. dILP2 contributes to the total body weight [26] and its secretion can be assessed by monitoring its accumulation into the cell bodies of IPCs. Accumulation is observed when dILP2 secretion is inhibited upon starvation [5]; (Figure 2A, B). Of the hits, 5 kinases caused significant dILP2 accumulation upon knockdown. These include Cdk12, Adck (CG3608), Pkc98E (Figure S1), as well as two Rio kinases, Rio1 and Rio2 (Figure 2A, B). Rio1 and Rio2 belong to a group of atypical kinases and they have a conserved role in ribosome maturation [27]–[30]. As ribosome biogenesis is a process tightly coupled to nutrient sensing [1], [12], we chose to further explore the role of Rio kinases in dILP secretion. We confirmed that, similarly to starvation, IPC-specific depletion of Rio kinases led to elevated *insulin-like receptor* (*inr*) gene expression in the larva (Figure 2C), which is an established readout for reduced peripheral insulin signalling [31]. If the inhibition of dILP2 secretion was due to impaired ribosome biogenesis, we predicted that depletion of other ribosomal genes would phenocopy this effect. Indeed, IPC-specific knockdown of a number of other ribosomal components or genes involved in various steps of ribosome biogenesis led to significantly reduced body size (Figure 2D). To further confirm that the dILP2 secretion phenotypes of Rio kinases were due to impaired ribosome biogenesis, we depleted ribosomal protein Rpl35A and ribosome assembly factor Tsr1 (CG7338) in the IPCs and observed a similar inhibition of dILP2 secretion in both cases (Figure 2E, F). The dILP2-Gal4 driver we used [4] displays activity in the salivary glands, in addition to IPCs. Depletion of Rio2 by a salivary gland specific Sgs3-Gal4 driver [32] caused no growth reduction, ruling out the possibility of nonspecific effects through the salivary glands (Figure S2).

TORC1 signalling regulates ribosome biogenesis gene expression in *Drosophila* in a context-dependent manner. In S2 cells, expression of ribosome biogenesis genes is TORC1-dependent [33], [34], while in the context of whole larvae, TOR mutants do not display significantly reduced expression of ribosome biogenesis genes [35]. In accordance with the earlier observations in whole larvae, inhibition of Raptor in the IPCs did not lead to inhibited dILP2 secretion (Figure S3). Transcription factor dMyc is a conserved master regulator of ribosome biogenesis [13], [36]. It transcriptionally regulates the expression of rRNAs as well as ribosomal proteins [36]. In *Drosophila* larvae ribosome biogenesis genes that are inhibited upon starvation are targets of dMyc [35]. Therefore we tested whether dMyc activity in the IPCs has an impact on dILP2 secretion. Indeed, RNAi-mediated depletion of dMyc prominently inhibited dILP2 secretion (Figure 2G, H) and led to reduced body weight (Figure 2I).

We wanted to further explore whether inhibition of ribosome biogenesis affects the secretion of other dILPs in the IPCs. Similarly to dILP2, secretion of dILP5 was inhibited upon Rio2 knockdown (Figure 2J, K). Inhibition of systemic growth often manifests as prolonged larval development, in addition to reduced body size. Indeed, depletion of Rio2 in the IPCs led to significant delay in pupation kinetics (Figure 2L). In conclusion, inhibition of ribosome biogenesis in the IPCs triggers a response that blocks dILP secretion and consequently leads to slower larval development and reduced body size.

### p53 mediates dILP2 accumulation upon inhibited ribosome biogenesis

**Figure 3:**
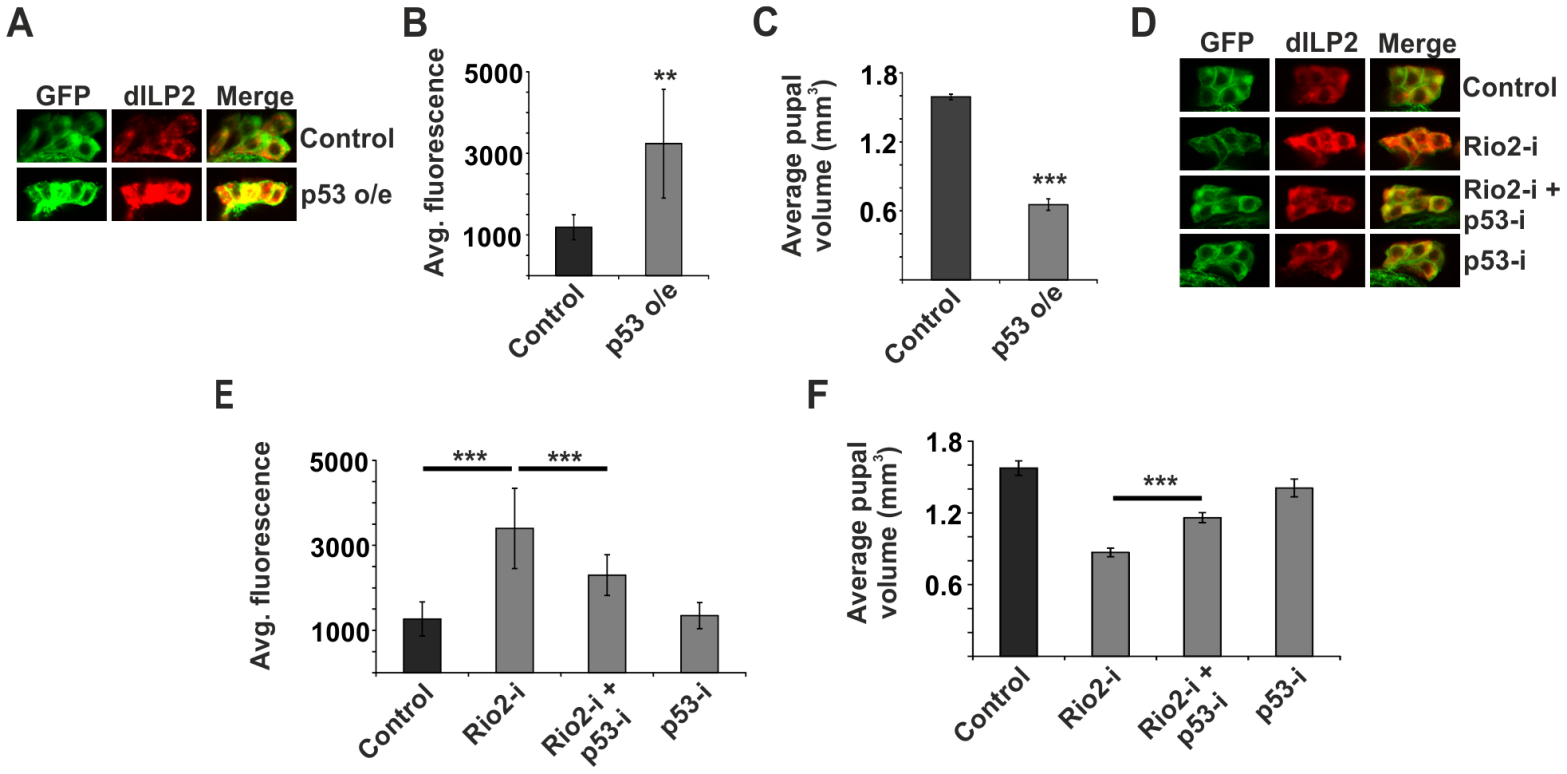
p53 mediates dILP2 accumulation upon inhibited ribosome biogenesis. (**A, B**) Overexpression of p53 in the IPCs is sufficient to inhibit dILP2 secretion. Error bars represent standard deviation, (N≥10 brains). (**C**) Overexpression of p53 in the IPCs leads to reduced pupal volume. Error bars represent standard deviation, (N = 3, 10 pupae/group). (**D, E**) Knockdown of p53 suppresses dILP2 accumulation following Rio2 depletion. Error bars represent standard deviation, (N≥10 brains). (**F**) Knockdown of p53 leads to partial rescue of pupal volume phenotype observed upon Rio2 depletion. Error bars represent standard deviation, (N = 3, 10 pupae/group). For all confocal images, IPCs are marked by GFP (green) and dILP2 is shown as red. **p<0.01, ***p<0.001 (Student's t-test).

How is the ribosome biogenesis pathway coupled to dILP secretion? We hypothesised that one of the ribosome biogenesis surveillance pathways might link secretion to the status of ribosome biogenesis in specialised secretory cells like the IPCs. As p53 is the best-established surveillance factor for the ribosome biogenesis pathway [14], [15], [37], we wanted to explore, whether it is involved in the regulation of dILP secretion. Indeed, overexpression of p53 in the IPCs revealed that p53 is sufficient to cause dILP2 accumulation (Figure 3A, B). To analyse the possible impact of transcriptional regulation, we measured *dilp2* mRNA levels by quantitative RT-PCR. p53 overexpression caused modest downregulation of *dilp2* mRNA levels (Figure S4), ruling out the possibility of transcriptional activation as a cause for p53-dependent dILP2 accumulation. To assess the contribution of p53-mediated dILP regulation on growth, we analyzed pupal volume, which is a sensitive means to measure changes in total body size [22]. In our hands, pupal volume data is consistent with the adult weight, but displays less random variation. In accordance with the immunofluorescence data suggesting inhibited dILP2 secretion, p53 overexpression in the IPCs strongly reduced the pupal volume (Figure 3C). In contrast, overexpression of p53 in the salivary glands caused no growth impairment (Figure S2). In sum, our data is consistent with the idea that p53 expression in the IPCs is sufficient to prevent dILP secretion and consequently inhibit tissue growth.

Next, we wanted to explore, whether the p53 would mediate the observed inhibition of dILP2 secretion upon disturbed ribosome biogenesis. This was the case, as depletion of p53 rescued the dILP2 accumulation upon Rio2 knockdown (Figure 3D, E) along with partial rescue of the growth impairment (Figure 3F). A similar rescue of growth was observed when p53 was depleted in combination with Rio1 (Figure S5). Moreover, a control RNAi targeting firefly Luciferase, but no endogenous genes, had no influence on the phenotypes of Rio2 RNAi in the IPCs (Figure S6), demonstrating that expression of an additional RNAi does not interfere with Rio2 RNAi activity.

### ERK7 mediates the inhibition of dILP secretion upon impaired ribosome biogenesis

**Figure 4:**
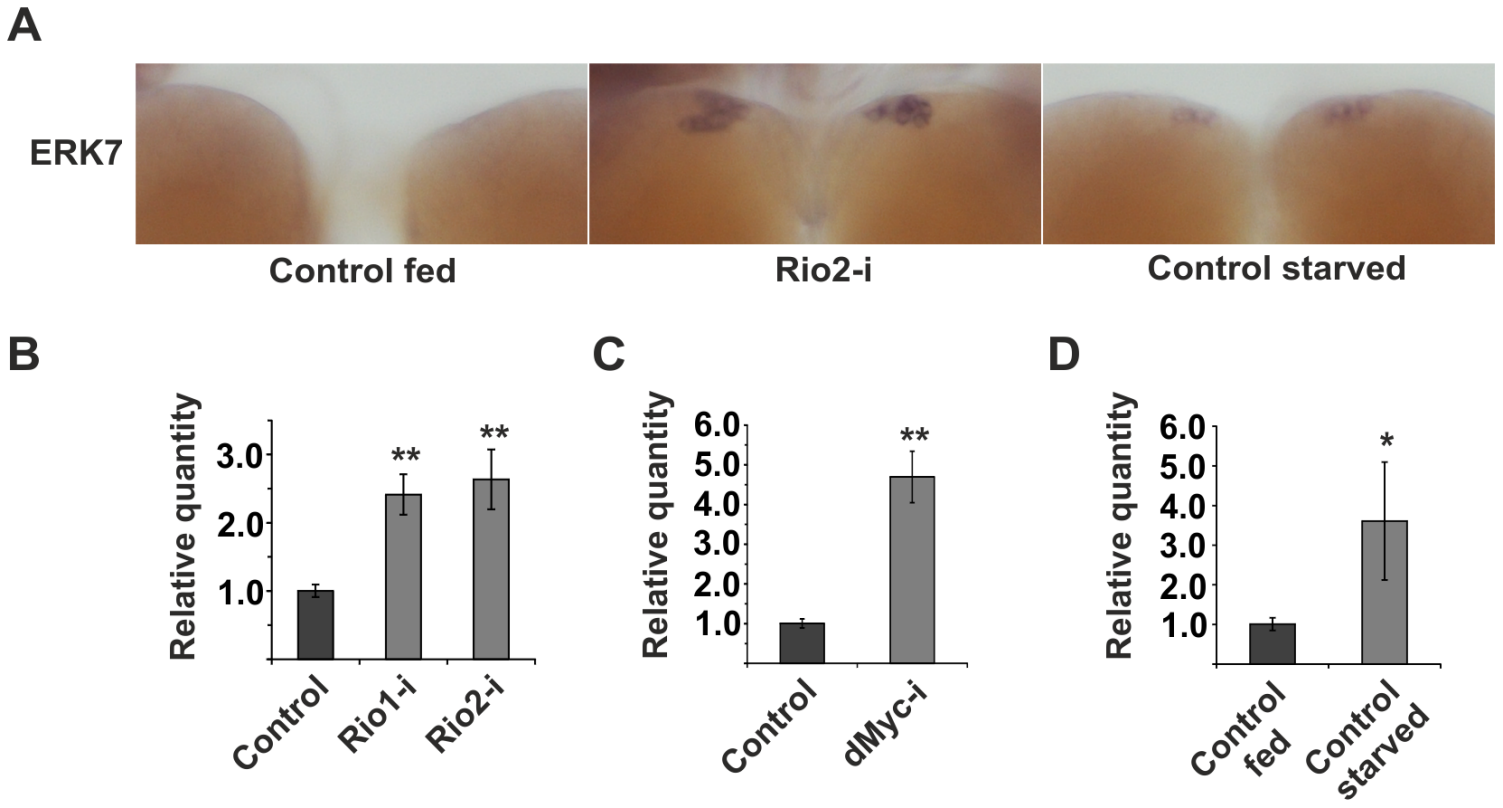
*erk7* mRNA levels are elevated upon impaired ribosome biogenesis or starvation. (**A**) ERK7 mRNA levels in the IPCs were detected by *in situ* hybridization upon IPC-specific knockdown of Rio2 (dILP2-Gal4) or starvation. (**B, C**) Quantitative RT-PCR analysis of relative *erk7* mRNA expression upon ubiquitous knockdown (Tub-G80^TS^; Tub-Gal4) of Rio1 and Rio2 (**B**), or dMyc (**C**) in whole larvae. (**D**) Relative *erk7* mRNA levels upon feeding and starvation in whole w^1118^ larvae. Error bars represent standard deviation (N = 3, ≥5 larvae/group). *actin* was used as an internal reference. *p<0.05, **p<0.01 (Student's t-test).

Identification of p53 as an essential inhibitor of dILP2 secretion implied that ribosome biogenesis regulates dILP secretion through an active surveillance mechanism, rather than through an indirect mechanism following reduced translation. To directly couple ribosome surveillance to secretion we turned our attention to known regulators of the secretory pathway. It was recently shown that an atypical MAP kinase called ERK7 (also known as ERK8 or MAPK15) inhibits secretion upon serum and amino acid starvation in *Drosophila* S2 cells [38]. ERK7 was shown to phosphorylate Sec16 and thus prevent its recruitment to ER exit sites, consequently preventing the export of the secretory cargo. Therefore, we wanted to explore, whether ERK7 is regulated in response to inhibited ribosome biogenesis. In order to do so, we used *in situ* hybridization of larval brain. *erk7* expression was undetectable in fed control larvae, whereas overexpression of transgenic ERK7 by dILP2-Gal4 led to a strong IPC-specific signal (Figure S7). As a negative control we used an *erk7* sense probe for the ERK7 overexpressing samples (Figure S7). After confirming the specificity of the *in situ* hybridization, we analysed *erk7* expression upon disturbed ribosome biogenesis. Intriguingly, we observed that *erk7* mRNA levels were clearly elevated in the IPCs following Rio2 depletion using dILP2-Gal4 (Figure 4A), implying that ERK7 is upregulated upon ribosome surveillance in the IPCs. As ribosome biogenesis is inhibited upon starvation [35], we hypothesized that starvation might also cause elevated expression of *erk7*, which indeed was the case (Figure 4A). Notably, in the context of brain tissue, the starvation-induced upregulation appeared specific to the IPCs, since *erk7* mRNA remained undetectable in other brain areas. To confirm the regulation of *erk7* mRNA by an independent method, we used quantitative RT-PCR on mRNA samples isolated from whole larvae. Disturbance of ribosome biogenesis by ubiquitous depletion of Rio1, Rio2 and Myc led to elevated *erk7* expression (Figure 4B, C). Similarly, starvation caused significant upregulation of *erk7* mRNA levels in the whole larval samples (Figure 4D). In sum, our data shows that *erk7* gene expression is elevated upon impaired ribosome biogenesis and starvation, suggesting a possible role for ERK7 in the regulation of dILP secretion during these conditions.

**Figure 5:**
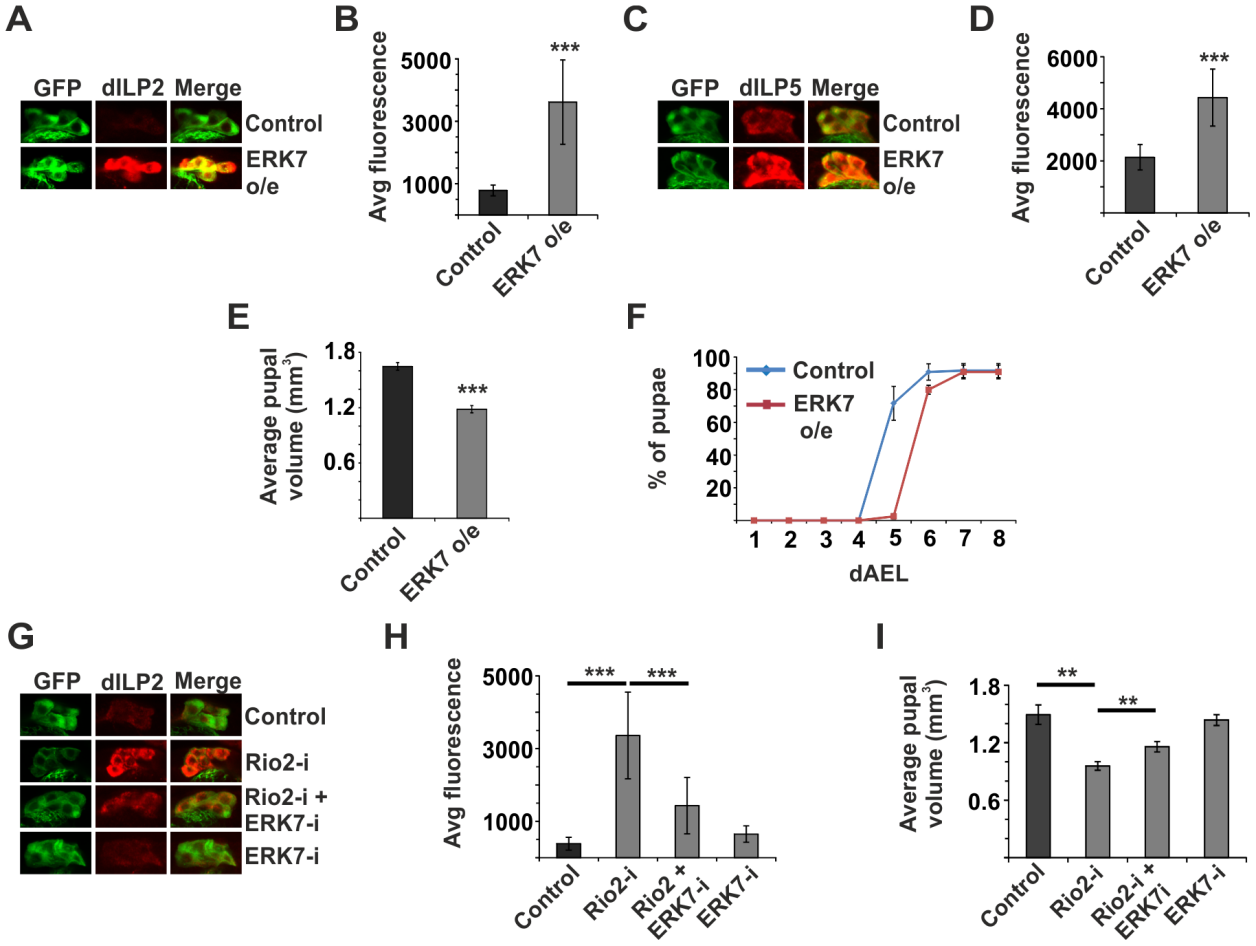
ERK7 mediates inhibition of dILP secretion upon disturbed ribosome biogenesis. Overexpression of ERK7 in the IPCs is sufficient to inhibit dILP2 (**A, B**) and dILP5 (**C, D**) secretion. Notably, dILP5 experiment was done together with Rio2 knockdown and the control dataset is the same as in Figure 2K. Error bars represent standard deviation, (N≥10 brains). (**E**) ERK7 overexpression in the IPCs results in smaller pupal volume. Error bars represent standard deviation, (N = 3, 10 pupae/group). (**F**) Overexpression of ERK7 in the IPCs leads to delayed pupation. Notably, this experiment was done together with Rio2 knockdown and the control dataset is the same as in Figure 2L. Error bars represent standard deviation, (N = 4, 30 larvae/group). (**G, H**) Accumulation of dILP2 in the cell bodies of IPCs upon Rio2 depletion is suppressed by simultaneous knockdown of ERK7. Error bars represent standard deviation (N≥10 brains). (**I**) Knockdown of ERK7 in the IPCs partially suppresses the small pupal size caused by Rio2 depletion. Error bars represent standard deviation (N = 3, 10 pupae/group). For all confocal images, IPCs are marked by GFP (green) and dILP2 or dILP5 is shown as red. **p<0.01, ***p<0.001 (Student's t-test).

To this end, we decided to explore the functional relationship between ribosome surveillance, ERK7 and dILP secretion in the IPCs. We analyzed transgenic flies that specifically overexpress ERK7 in the IPCs (Figure S7). Indeed, ERK7 overexpression led to prominent accumulation of dILP2 and dILP5 in the IPCs (Figure 5A–5D). Consistently, ERK7 overexpression in the IPCs, but not in salivary glands, led to reduced pupal volume (Figure 5E and Figure S2) as well as delayed pupation (Figure 5F). To rule out the possibility that the ERK7-dependent dILP accumulation was due to elevated transcription, we used quantitative RT-PCR to measure *dilp2* and *dilp5* mRNA levels, which remained unchanged upon ERK7 overexpression (Figure S8). Thus, we conclude that elevated ERK7 expression is sufficient to inhibit dILP secretion cell autonomously in the IPCs. We also performed genetic epistasis experiments to test if ERK7 is essential to inhibit dILP2 secretion upon disturbed ribosome biogenesis. Indeed, ERK7 RNAi efficiently suppressed the dILP2 accumulation caused by Rio2 knockdown (Figure 5G, H). Similar suppression was observed in the case of dILP2 accumulation following dMyc depletion (Figure S9). Consistent with the effects on dILP2 secretion, IPC-specific depletion of ERK7 partially suppressed the impaired growth upon knockdown of Rio2, while having no effect on body size in the wild type background (Figure 5I).

### ERK7 acts downstream of p53 in nutrient responsive regulation of dILP2 secretion

**Figure 6:**
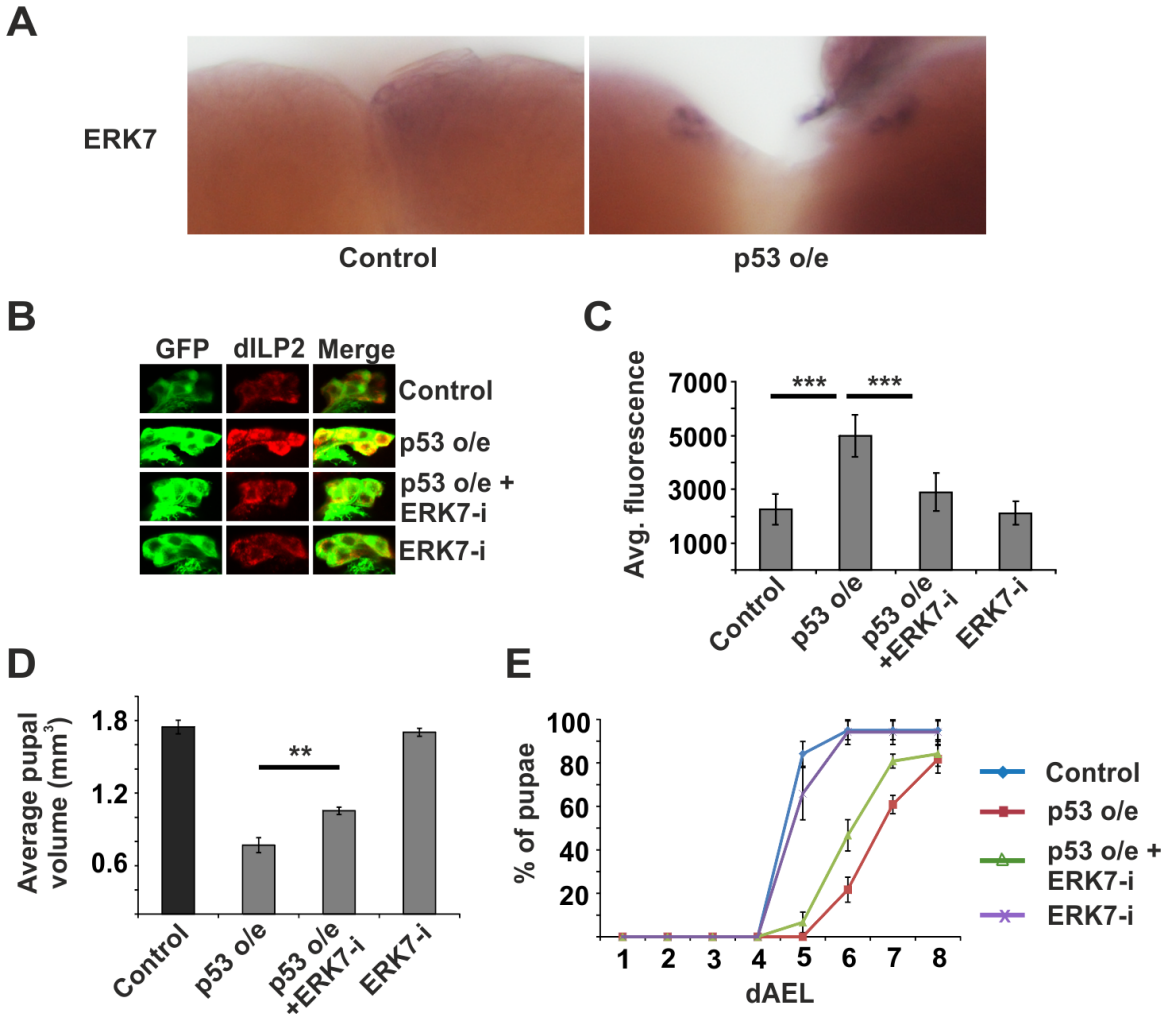
ERK7 acts downstream of p53. (**A**) Overexpression of p53 in the IPCs (dILP2-Gal4) leads to increased *erk7* mRNA levels as detected by *in situ* hybridization. (**B, C**) Knockdown of ERK7 suppresses dILP2 accumulation following p53 overexpression. Error bars represent standard deviation, (N≥10 brains). (**D**) Knockdown of ERK7 in the IPCs rescues the small pupal size caused by p53 overexpression. Error bars represent standard deviation (N = 3, 10 pupae/group). (**E**) ERK7 depletion in the IPCs partially rescues the developmental delay caused by p53 overexpression. Error bars represent standard deviation (N = 4, 30 larvae/group). **p<0.01, ***p<0.001 (Student's t-test).

As we had observed that both p53 and ERK7 were essential for the inhibition of dILP2 secretion upon impaired ribosome biogenesis, we wanted to explore whether they belong to the same pathway. We hypothesized that ERK7 might be a downstream effector of p53. Supporting this idea, we observed that overexpression of wild type p53 in the IPCs led to elevated expression of *erk7* mRNA as detected by *in situ* hybridization (Figure 6A). To further test the possible relevance of the p53-mediated regulation of *erk7* in the IPCs, we performed genetic epistasis experiments. Indeed, depletion of ERK7 efficiently suppressed the dILP2 accumulation upon p53 overexpression (Figure 6B, C). Consistently, ERK7 RNAi also partially suppressed the reduction of pupal volume caused by p53 overexpression (Figure 6D) along with the delay in pupation (Figure 6E). Notably, the suppression by ERK7 RNAi was only partial, leaving open the possibility for a parallel, ERK7-independent, mechanism. In sum, our data implies that p53 and ERK7 belong to the same pathway and ERK7 acts as a downstream effector of p53 in inhibiting dILP2 secretion.

**Figure 7:**
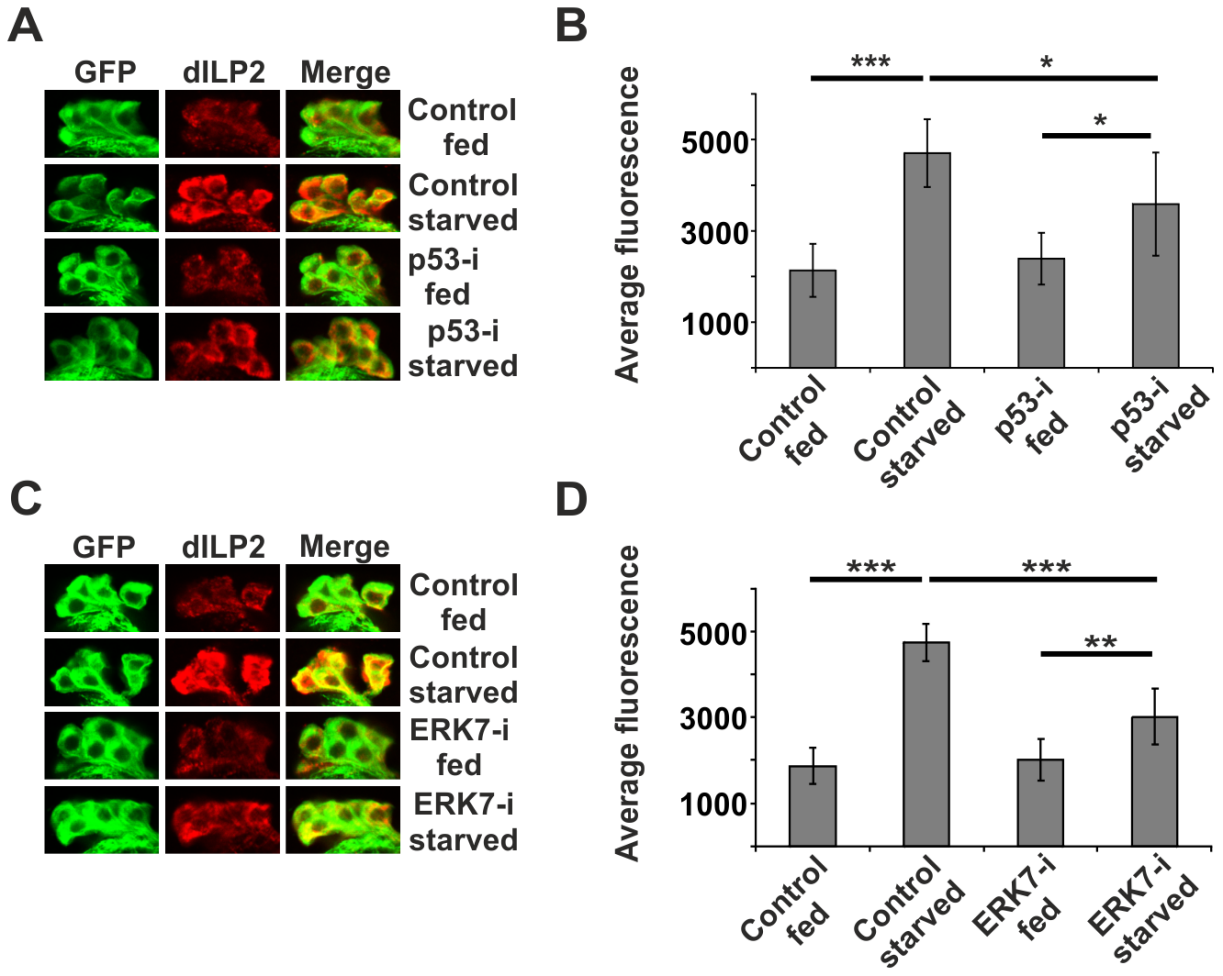
p53 and ERK7 regulate dILP2 secretion upon starvation. Knockdown of p53 (**A, B**) or ERK7 (**C, D**) in the IPCs partially suppresses dILP2 accumulation upon starvation. Error bars represent standard deviation, (N≥10 brains). Third instar non-wandering larvae were starved for 15 h on PBS, 1% sucrose, 0.4% agar. IPCs are labelled by GFP (green) and dILP2 is shown as red. *p<0.05, **p<0.01, ***p<0.001 (Student's t-test).

As ribosome biogenesis is dependent on nutrients [1], [12], [35], and *erk7* expression was elevated upon starvation (Figure 4A, D), we postulated that perhaps the p53- and ERK7-dependent ribosome surveillance response contributes to dILP accumulation in the IPCs upon starvation. This proved to be the case, as inhibition of either p53 (Figure 7A, B; Figure S10) or ERK7 (Figure 7C, D) in the IPCs partially reduced the accumulation of dILP2 following starvation of the larvae. While we cannot rule out the possibility that the knockdown of p53 and ERK7 affects dILP2 translation in this setting, the most parsimonious explanation is that p53 and ERK7 contribute to dILP2 secretion upon starvation.

## Discussion

Here we report a novel cell-autonomous control mechanism for dILP secretion. Specifically, we conclude that i) inhibition of ribosome biogenesis in the IPCs at any level tested, including ribosomal gene expression (Myc), ribosome maturation (Rio1, Rio2, Tsr1) or by introducing imbalance of ribosomal components (Rpl35A), triggers a response to inhibit dILP secretion, ii) this inhibitory response is dependent on p53, a known surveillance factor for ribosome biogenesis, iii) a downstream effector of this ribosome surveillance pathway is protein kinase ERK7, as *erk7* mRNA levels are elevated upon inhibited ribosome biogenesis and p53 activation and *erk7* is essential to inhibit dILP secretion in both conditions, iv) the ribosome surveillance mechanism discovered here likely contributes to starvation-induced inhibition of dILP secretion. These findings significantly broaden the view about the regulatory functions of the ribosome surveillance pathways, which have been mainly explored at the level of proliferating cells. Ribosome biogenesis serves as the key determinant of cell autonomous growth control and it is finely tuned to match with the cellular nutrient and energy status [1], [12]. Coupling dILP secretion to the ribosome biogenesis pathway is an elegant mechanism for multicellular animals to synchronize the hormonal growth control with the local cell autonomous regulation of growth. Comparable to our finding in the IPCs, inhibition of ribosome biogenesis in the fat body leads to a block of dILP secretion in the IPCs through an unknown humoral mechanism [22], [24], [39]. Linking ribosome biogenesis to growth control through parallel mechanisms likely provides a robust regulatory network to tune down systemic growth signals whenever any region of the body experiences nutrient deprivation. This synchronization is likely important to maintain balanced growth throughout the spectrum of dietary conditions. Future studies should be aimed to explore the possible interrelationship between the fat body-derived signals and the cell-autonomous mechanism discovered here.

Our findings highlight that the p53-mediated ribosome surveillance pathway can serve highly cell type-specific functions *in vivo*. This is interesting when considering human ribosomopathies, genetic diseases caused by impaired ribosome biogenesis manifesting with a wide spectrum of tissue-specific defects. One of the ribosomopathies, Shwachman-Diamond syndrome (SDS), is manifested with a failure in pancreatic function [20]. A mouse model of SDS displays general preservation of ductal and endocrine compartments, but reduced amount of zymogen granules. Moreover, SDS mutant mice have reduced glucose tolerance, suggesting compromised endocrine function [40]. It will be interesting to learn whether p53 and ERK7 act as mediators of the secretion-related defects observed in SDS.

Compared to other members of the MAP kinase family, ERK7 has remained relatively poorly characterized [41]. Earlier studies in mammalian and *Drosophila* cells have shown that ERK7 protein levels are actively regulated at the level of protein degradation [38], [42]. In mammals, an increase in ERK7 levels leads to autophosphorylation and consequent activation [43], [44]. Consistent with the idea that ERK7 is mainly regulated through abundance, we observed that elevated ERK7 expression had a prominent impact in the function of IPCs. Interestingly, earlier studies have linked ERK7 function to growth regulation by showing that ERK7 protein is stabilized by serum and amino acid starvation [38]. Our data provides evidence that impaired ribosome biogenesis as well as starvation increases the expression of *erk7* mRNA revealing a novel regulatory level for ERK7 function. In addition to the conditions explored here, ERK7 expression levels increase towards the end of larval development when growth is ceased [45]. It will be interesting to learn further how ERK7 expression is regulated and whether ERK7 has a function in tissue growth control beyond its role in the IPCs. Earlier studies in cell culture have shown evidence that ERK7 regulates cancer cell proliferation and autophagy [46], [47], suggesting that ERK7 may have a broader role in the regulation of tissue growth.

## Materials and Methods

### Fly stocks and husbandry

RNAi lines were from Vienna *Drosophila* RNAi Center (VDRC), Bloomington *Drosophila* Stock Center (BDSC) and National Institute of Genetics (NIG), Japan. Identities of RNAi lines and transgenes are listed in Table S2. Driver lines used: dILP2-Gal4, UAS-GFP [4] for all IPC specific experiments, Tub-G80^TS^; Tub-Gal4 (temperature sensitive ubiquitous driver) for ubiquitous expression of RNAi. Sgs3-Gal4 (BDSC#6870) was used for salivary gland specific experiments [32]. w^1118^ crossed to respective driver lines was used as control in all experiments. For generating UAS-ERK7-V5-His, ERK7 cDNA was amplified by PCR from pMT-ERK7-V5-HisB [38], cloned into KpnI site of the pUAST-attB vector and confirmed by sequencing. The transgene was directed to the attP landing site at 22A2. Transgenic flies were constructed by BestGene Inc.

Flies were grown on medium containing agar 0.6% (w/v), semolina 3.2% (w/v), malt 6.5% (w/v), dry baker's yeast 1.8% (w/v), propionic acid 0.7% (v/v) and Nipagin (methylparaben) 2.4% (v/v). For starvation, third instar larvae were placed on PBS, 1% sucrose, 0.4% agar for approximately 15 hours.

### *Drosophila* relative weight determination

To normalize variation between vials due to growth conditions, we determined the relative weight. UAS-RNAi flies were crossed to dILP2-Gal4, UAS-GFP/CyO flies and progeny were allowed to develop at 29°C. Four days after adult emergence, male flies were weighed in groups (≥10 flies/group, N≥3), using a precision balance (Mettler). The mean weight of the flies expressing both dILP2-Gal4 and RNAi was divided by the mean weight of control flies (not expressing the dILP2-GAL4) from the same vial.

### Measurement of pupal volume

UAS-RNAi flies were crossed to dILP2-Gal4, UAS-GFP/Cyo flies and allowed to lay eggs at +25°C. GFP positive L1 larvae were collected (30 per vial) 24 hours after egg deposition (AED) and grown at +29°C until pupation. Length and width of pupae were measured using ProgRes CapturePro 2.8.8 program (Jenoptik). Volumes of the pupae were calculated using the following formula: 4/3π(L/2)(W/2)^2^ (L, length; W, width).

### Immunostainings and antibodies

Brains were dissected from 3rd instar wandering larvae, fixed in 4% formaldehyde-PBS for 30 min at RT and washed in PBT buffer (0.3% Triton X 100 in PBS). Following blocking for 2 h at RT in 5% bovine serum albumin (BSA)/PBT, samples were incubated with respective primary antibodies at 4°C o/n. Samples were washed three times (15 min each) with PBT and incubated with respective secondary antibodies for 2 h at RT. After three washes in PBT, brains were mounted in Vectashield Mounting Medium (Vector laboratories). Fluorescence images were acquired using a Leica TCS SP5 MP SMD FLIM confocal laser scanning microscope. Antibodies used: rat anti-dILP2 [5], rabbit anti-dILP2 [48], guinea pig anti-dILP2 (see below), rabbit anti-dILP5 (5), anti-rat Alexa fluor 633 (Invitrogen), anti-rabbit Alexa fluor 633 (Invitrogen) and anti-guinea pig Alexa fluor 633 (Invitrogen). Confocal images of each IPC cluster were taken using the same scan and laser power settings. Total signal from each cluster was quantified using ImageJ software (NIH). Representative images were cropped and processed identically for all the samples of every experiment.

### dILP2 antibody generation

Two synthetic peptides (PHKRAMPGADSDLDA) and (AEVRRRTRQRQGI) corresponding to amino acid sequence of dILP2 from 49–63 and 102–114 amino acids respectively were produced by Storkbio Ltd. (Estonia). These peptides were mixed and used as immunogens to raise polyclonal antibodies in Guinea pigs by Storkbio Ltd.

### RNA extraction

RNA extraction from larval brains: Equal numbers of L1 larvae were collected and grown at 29°C. 10 brains from third instar non-wandering larvae (approx. 85 hours AED) were dissected and RNA was extracted using Nucleospin RNA XS kit (Macherey-Nagel) according to the manufacturer's protocol. RNA extraction from whole larvae: equal numbers of L1 larvae were collected and grown at 29°C for 72 h prior to harvesting (96 h AED). ≥5 third instar larvae per sample in minimum of three replicates were homogenized and RNA was extracted using Nucleospin RNA II kit (Macherey-Nagel) according to the manufacturer's protocol.

### Quantitative RT-PCR

RNA was reverse transcribed using RevertAid H Minus First Strand cDNA Synthesis Kit (Thermo Scientific). qPCR was performed using Maxima SYBR Green qPCR Master Mix (2X) (Fermentas) with Light cycler 480 Real-Time PCR System (Roche). At least three biological replicates were used for each genotype and at least three technical replicates were used for each biological replicate. Primers for quantitative RT-PCR are presented as Table S3.

### *In situ* hybridization

In situ hybridization was performed as described previously [49]. Briefly, antisense and sense probes were synthetized by T3/T7 RNA polymerase-dependent *in vitro* transcription reaction (Promega) in the presence of digoxigenin-UTP (Roche). Mid-3^rd^ instar larval tissues were fixed using 4% formaldehyde and they were hybridized with the labeled probes for 16 h at 55°C. The hybridization was visualized by using alkaline phosphatase-conjugated anti-digoxigenin Fab fragments (1∶3,000; Roche) in the presence of NBT and BCIP substrates (Promega).

Primers for the *erk7* probes were as follows:

5′-TAATACGACTCACTATAGGGCTCAAGAGCGACGCATTCAA-3′

5′-ATTAACCCTCACTAAAGGGATACTGGTCCACGTCGTAGCG-3′

## Supporting Information

Figure S1 Kinases influencing dILP2 secretion. (**A, B**) Knockdown of Adck, Cdk12 or Pkc98E inhibits dILP2 secretion from IPCs. Error bars represent standard deviation (N≥10). IPCs are labelled by GFP (green) and dILP2 is shown as red. *p<0.05, **p<0.01 (Student's t-test).
(TIF)

Figure S2 Rio2 knockdown or overexpression of ERK7 and p53 in the salivary glands does not affect growth. Pupal volumes following depletion of Rio2 or overexpression of either ERK7 or p53 in salivary glands using Sgs3-Gal4. Error bars represent standard deviation (N = 3, 10 pupae/group).
(TIF)

Figure S3 Depletion of Raptor in the IPCs does not inhibit dILP2 secretion. (**A, B**) Knockdown Raptor in IPCs does not lead to significant accumulation of dILP2 in cell bodies of IPCs. Error bars represent standard deviation (N≥10). IPCs are labelled by GFP (green) and dILP2 is shown as red. ns: p>0.05 (Student's t-test).
(TIF)

Figure S4 p53 overexpression in the IPCs modestly downregulates *dilp2* mRNA. *dilp2* mRNA levels from larval brain RNA were determined using quantitative RT-PCR. Error bars represent standard deviation (N = 3, 10 brains/group). GAPDH was used as an internal reference. *p<0.05 (Student's t-test).
(TIF)

Figure S5 Knockdown of p53 leads to rescue of reduced pupal volume observed upon Rio1 depletion. Error bars represent standard deviation, (N = 4, 10 pupae/group). **p<0.01 (Student's t-test).
(TIF)

Figure S6 RNAi against firefly Luciferase does not suppress Rio2-RNAi phenotypes. (**A, B**) Accumulation of dILP2 in the cell bodies of IPCs upon Rio2 depletion is not suppressed by RNAi against firefly Luciferase (not targeting any endogenous gene). Error bars represent standard deviation (N≥10 brains). IPCs are marked by GFP (green) and dILP2 is shown as red. (**C**) Luciferase RNAi in the IPCs does not rescue the small pupal size caused by Rio2 depletion. Error bars represent standard deviation (N = 3, 10 pupae/group). ***p<0.001, ns: p>0.05 (Student's t-test).
(TIF)

Figure S7 Controls for *in situ* hybridization. Endogenous *erk7* mRNA remains undetectable in the brains of fed control animals (dILP2-Gal4 x w^1118^), while IPC-specific overexpression of transgenic *erk7* allows detection of strong IPC-specific staining. *erk7* mRNA expression is detected using the antisense probe, but not using the sense probe (negative control).
(TIF)

Figure S8 Transcription of *dilp2* and *dilp5* is not affected by ERK7 overexpression. Transgenic ERK7 was overexpressed using dILP2-Gal4 driver. *dilp2* and *dilp5* mRNA levels were determined from brain RNA samples using quantitative RT-PCR. Error bars represent standard deviation (N = 3, 10 brains/group). GAPDH was used as an internal reference. ns: p>0.05 (Student's t-test).
(TIF)

Figure S9 ERK7 mediates inhibition of dILP2 secretion upon dMyc depletion. (**A, B**) Accumulation of dILP2 in the cell bodies of IPCs upon dMyc knockdown is suppressed by simultaneous knockdown of ERK7. Error bars represent standard deviation (N≥10 brains). IPCs are marked by GFP (green) and dILP2 is shown as red. ***p<0.001 (Student's t-test).
(TIF)

Figure S10 p53 contributes to regulation of dILP2 secretion upon starvation. (**A, B**) Expression of dominant negative p53 in the IPCs suppresses dILP2 accumulation following starvation. Error bars represent standard deviation, (N≥10 brains). **p<0.01, ***p<0.001 (Student's t-test).
(TIF)

Table S1 Primary screening data of the kinome-wide screen with RNAi expressed specifically in the IPCs.
(XLSX)

Table S2 Identities of RNAi lines and transgenes used in the study.
(XLSX)

Table S3 Sequences of primers used in the quantitative RT-PCR experiments.
(DOCX)
