# Supplementary figures and images for: p53- and ERK7-Dependent Ribosome Surveillance Response Regulates Drosophila Insulin-Like Peptide Secretion
Source: PLoS Genet. 2014 Nov 13;10(11):e1004764. doi: 10.1371/journal.pgen.1004764 (PMC4230838; doi:10.1371/journal.pgen.1004764)

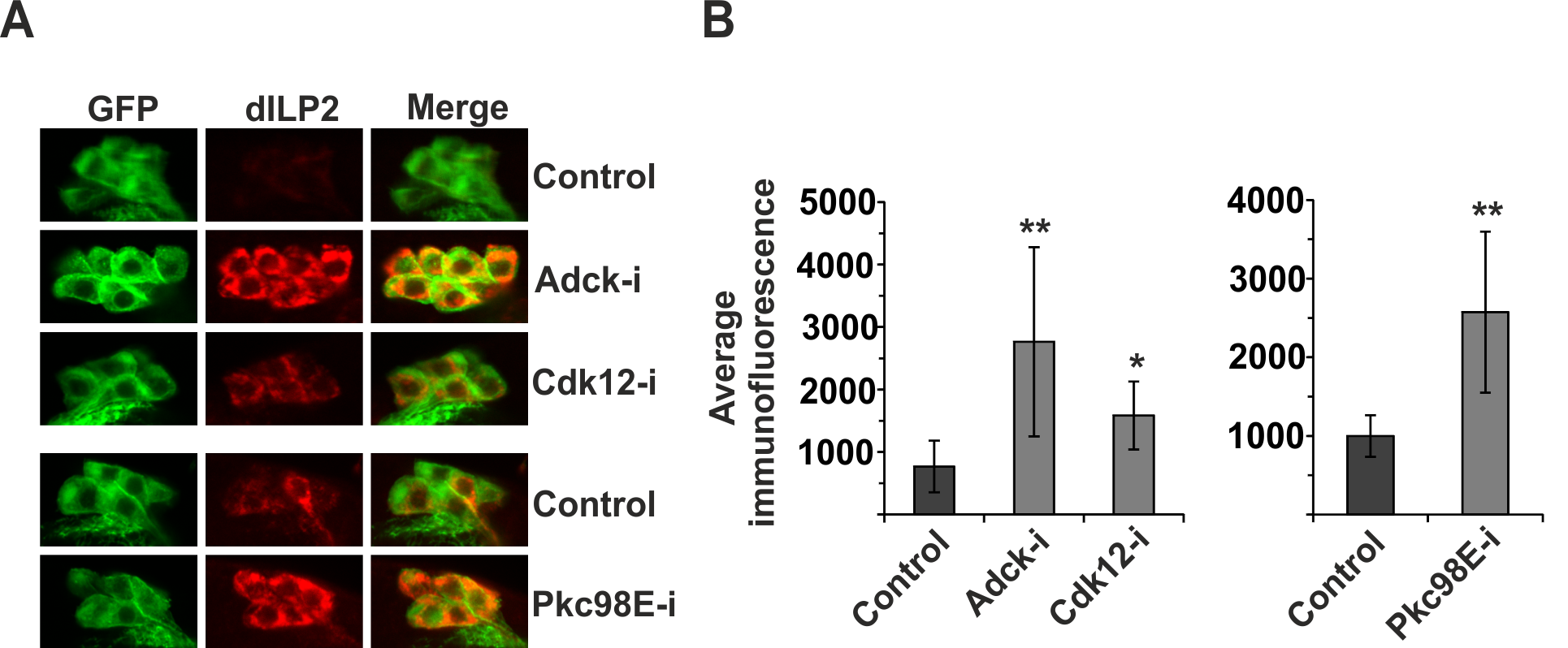

Supplement: Figure S1 — Kinases influencing dILP2 secretion. (A, B) Knockdown of Adck, Cdk12 or Pkc98E inhibits dILP2 secretion from IPCs. Error bars represent standard deviation (N≥10). IPCs are labelled by GFP (green) and dILP2 is shown as red. *p<0.05, **p<0.01 (Student's t-test). (TIF) [file pgen.1004764.s001.tif]

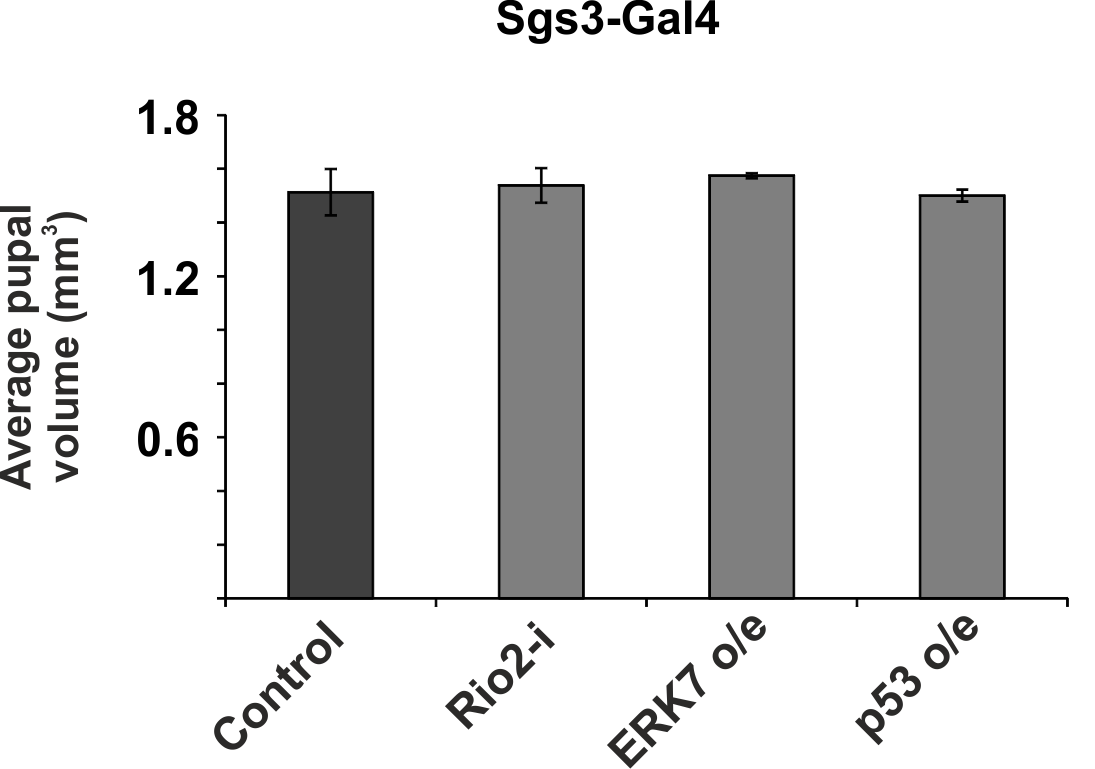

Supplement: Figure S2 — Rio2 knockdown or overexpression of ERK7 and p53 in the salivary glands does not affect growth. Pupal volumes following depletion of Rio2 or overexpression of either ERK7 or p53 in salivary glands using Sgs3-Gal4. Error bars represent standard deviation (N = 3, 10 pupae/group). (TIF) [file pgen.1004764.s002.tif]

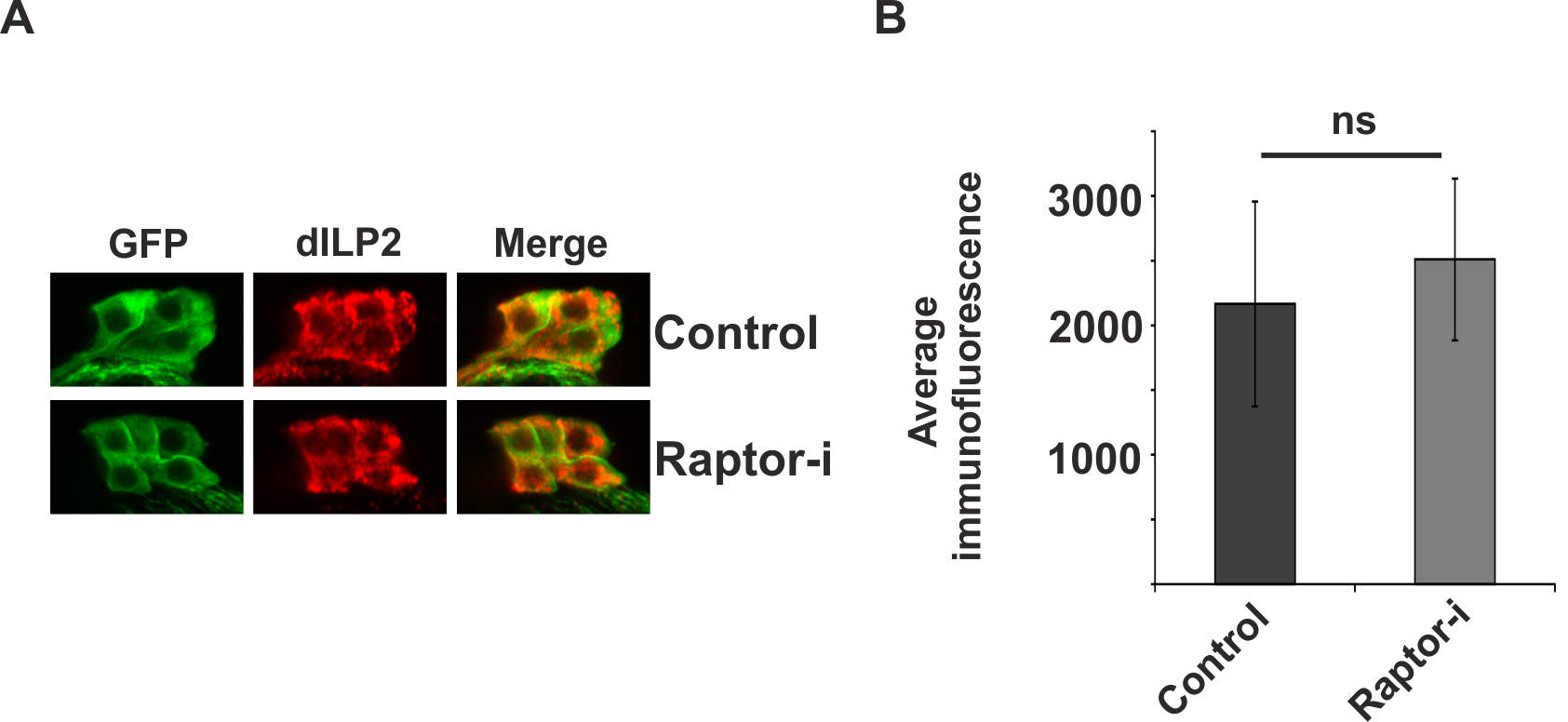

Supplement: Figure S3 — Depletion of Raptor in the IPCs does not inhibit dILP2 secretion. (A, B) Knockdown Raptor in IPCs does not lead to significant accumulation of dILP2 in cell bodies of IPCs. Error bars represent standard deviation (N≥10). IPCs are labelled by GFP (green) and dILP2 is shown as red. ns: p>0.05 (Student's t-test). (TIF) [file pgen.1004764.s003.tif]

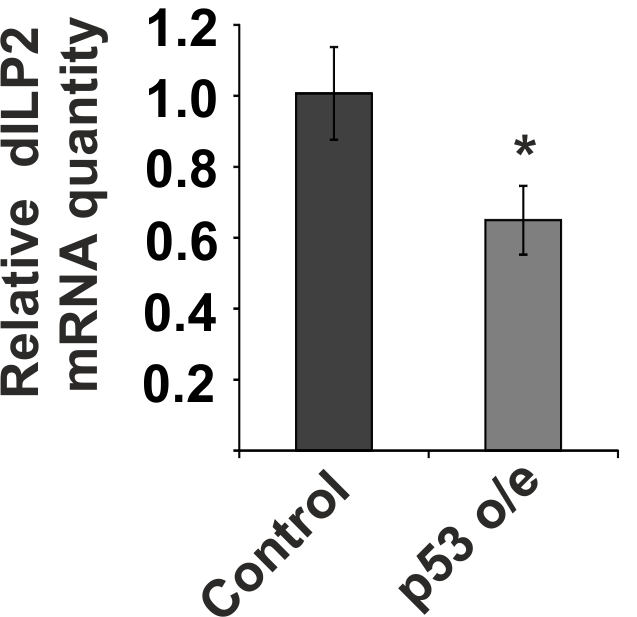

Supplement: Figure S4 — p53 overexpression in the IPCs modestly downregulates dilp2 mRNA. dilp2 mRNA levels from larval brain RNA were determined using quantitative RT-PCR. Error bars represent standard deviation (N = 3, 10 brains/group). GAPDH was used as an internal reference. *p<0.05 (Student's t-test). (TIF) [file pgen.1004764.s004.tif]

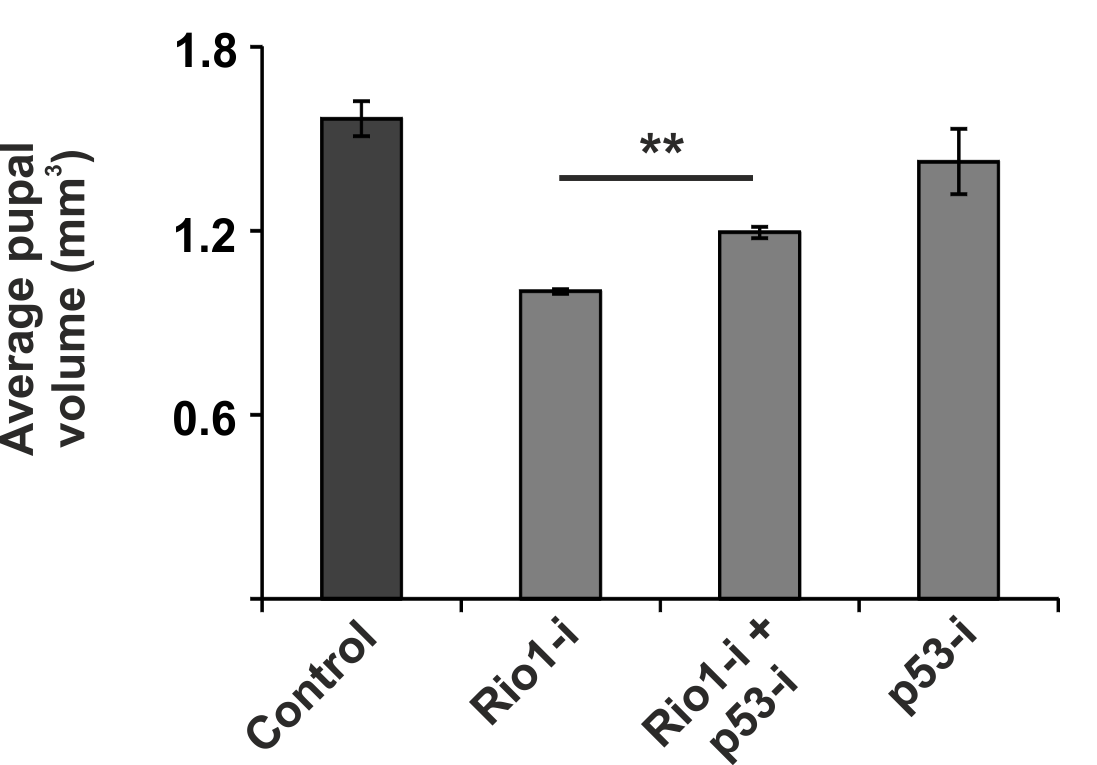

Supplement: Figure S5 — Knockdown of p53 leads to rescue of reduced pupal volume observed upon Rio1 depletion. Error bars represent standard deviation, (N = 4, 10 pupae/group). **p<0.01 (Student's t-test). (TIF) [file pgen.1004764.s005.tif]

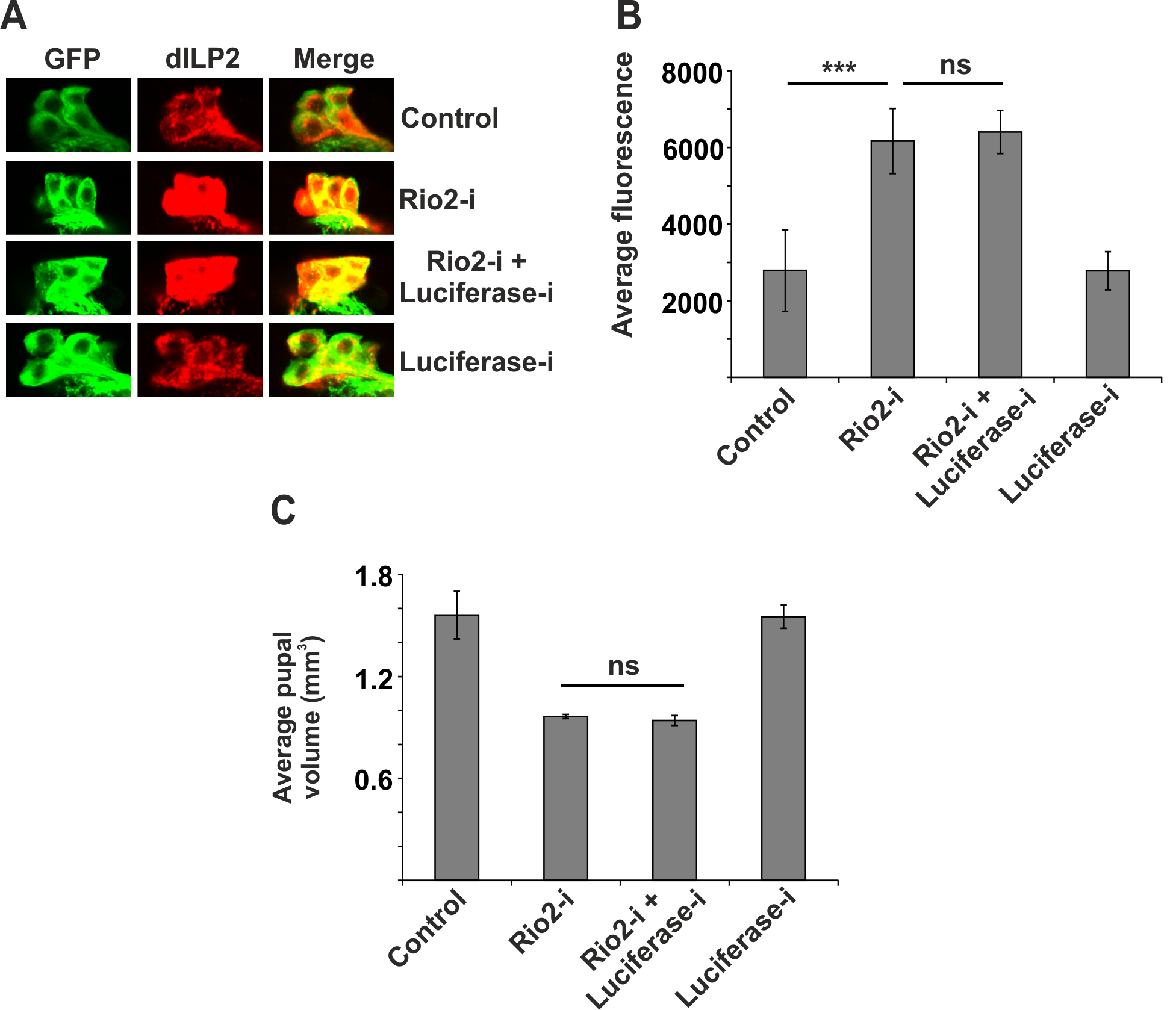

Supplement: Figure S6 — RNAi against firefly Luciferase does not suppress Rio2-RNAi phenotypes. (A, B) Accumulation of dILP2 in the cell bodies of IPCs upon Rio2 depletion is not suppressed by RNAi against firefly Luciferase (not targeting any endogenous gene). Error bars represent standard deviation (N≥10 brains). IPCs are marked by GFP (green) and dILP2 is shown as red. (C) Luciferase RNAi in the IPCs does not rescue the small pupal size caused by Rio2 depletion. Error bars represent standard deviation (N = 3, 10 pupae/group). ***p<0.001, ns: p>0.05 (Student's t-test). (TIF) [file pgen.1004764.s006.tif]

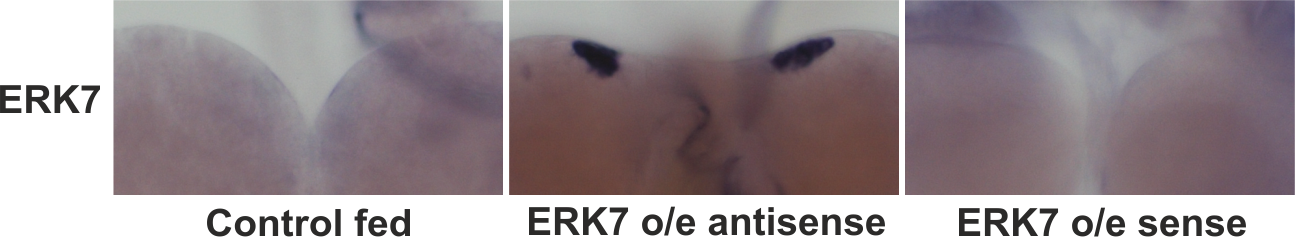

Supplement: Figure S7 — Controls for in situ hybridization. Endogenous erk7 mRNA remains undetectable in the brains of fed control animals (dILP2-Gal4 x w1118), while IPC-specific overexpression of transgenic erk7 allows detection of strong IPC-specific staining. erk7 mRNA expression is detected using the antisense probe, but not using the sense probe (negative control). (TIF) [file pgen.1004764.s007.tif]

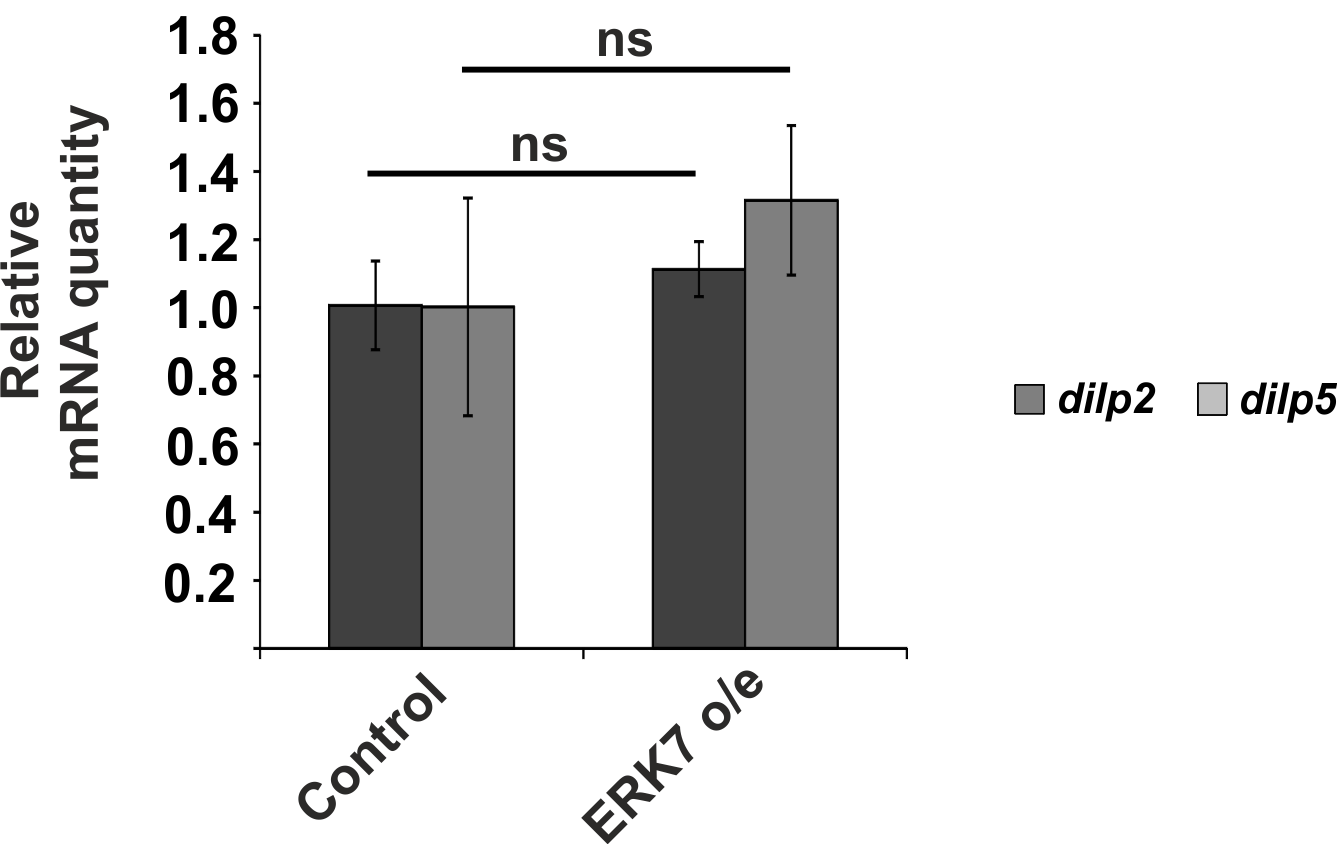

Supplement: Figure S8 — Transcription of dilp2 and dilp5 is not affected by ERK7 overexpression. Transgenic ERK7 was overexpressed using dILP2-Gal4 driver. dilp2 and dilp5 mRNA levels were determined from brain RNA samples using quantitative RT-PCR. Error bars represent standard deviation (N = 3, 10 brains/group). GAPDH was used as an internal reference. ns: p>0.05 (Student's t-test). (TIF) [file pgen.1004764.s008.tif]

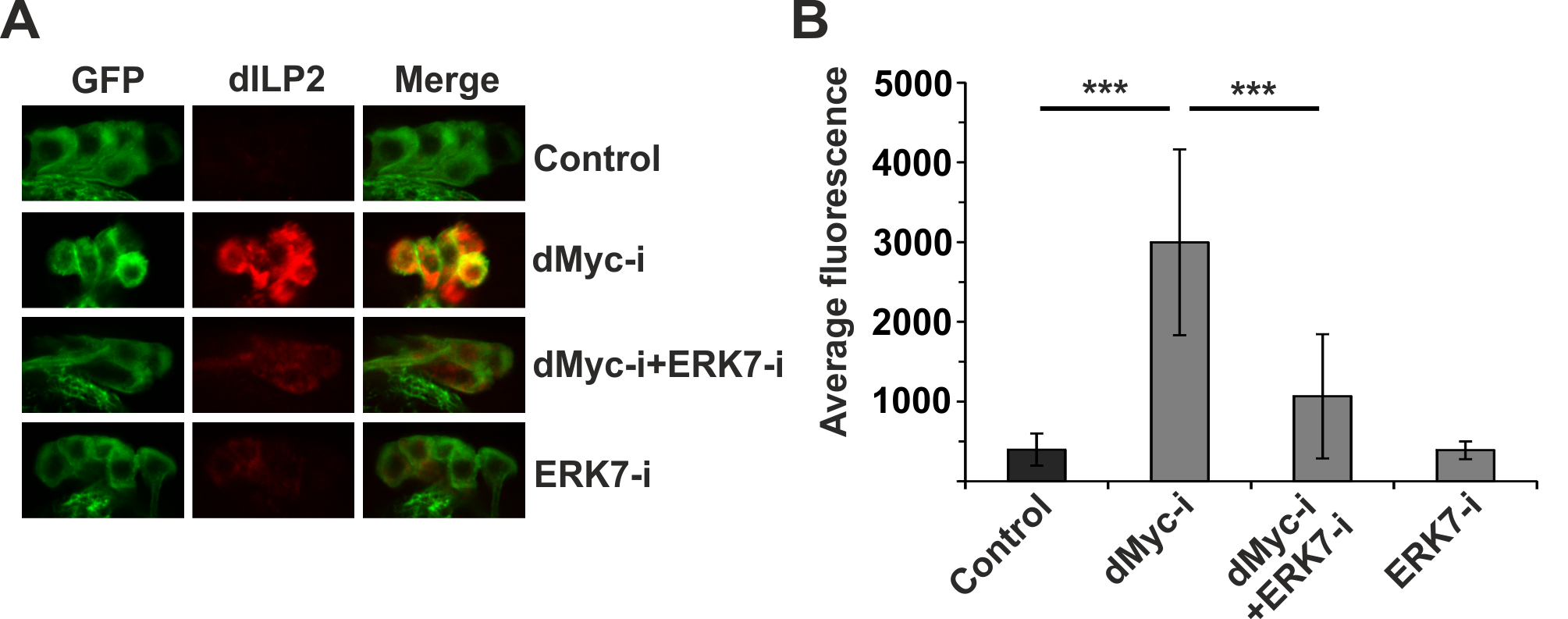

Supplement: Figure S9 — ERK7 mediates inhibition of dILP2 secretion upon dMyc depletion. (A, B) Accumulation of dILP2 in the cell bodies of IPCs upon dMyc knockdown is suppressed by simultaneous knockdown of ERK7. Error bars represent standard deviation (N≥10 brains). IPCs are marked by GFP (green) and dILP2 is shown as red. ***p<0.001 (Student's t-test). (TIF) [file pgen.1004764.s009.tif]

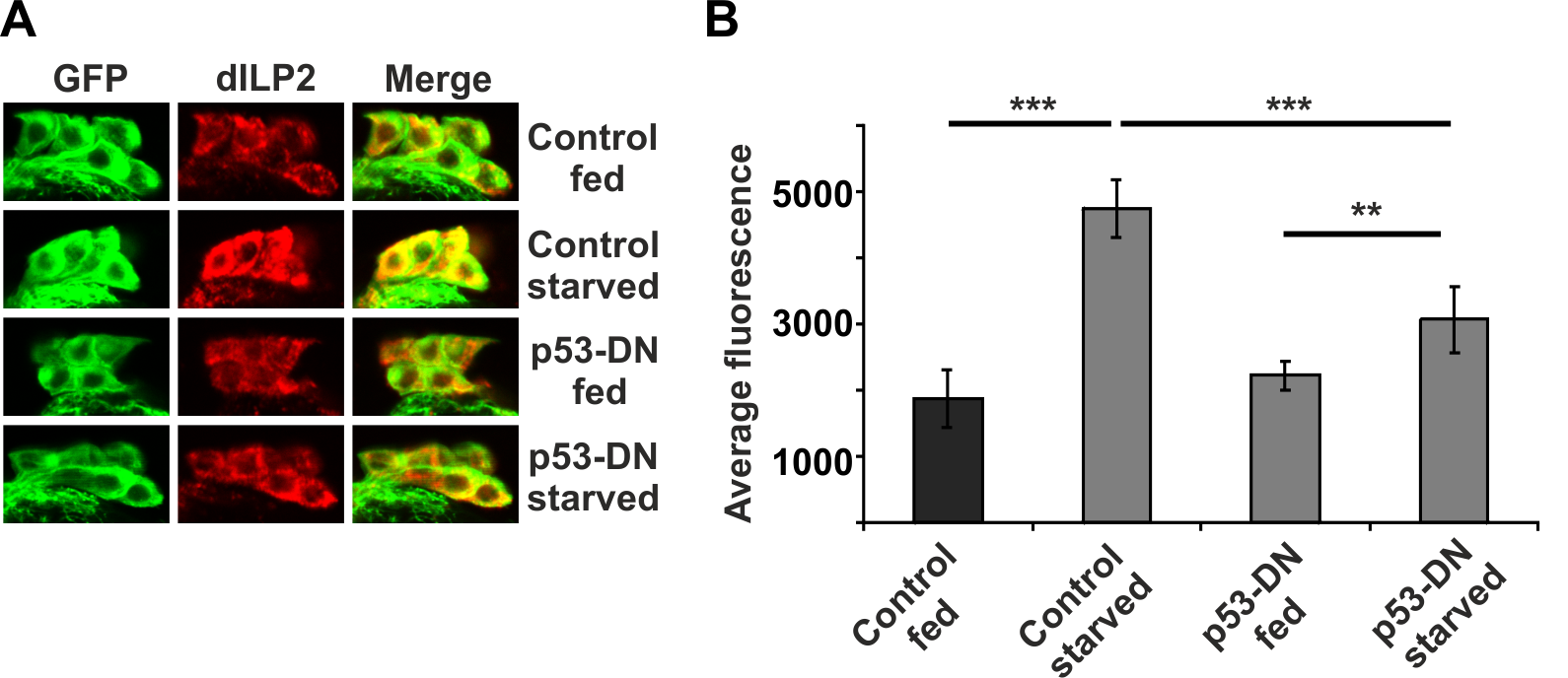

Supplement: Figure S10 — p53 contributes to regulation of dILP2 secretion upon starvation. (A, B) Expression of dominant negative p53 in the IPCs suppresses dILP2 accumulation following starvation. Error bars represent standard deviation, (N≥10 brains). **p<0.01, ***p<0.001 (Student's t-test). (TIF) [file pgen.1004764.s010.tif]
