# Supplementary material for: p53- and ERK7-Dependent Ribosome Surveillance Response Regulates Drosophila Insulin-Like Peptide Secretion
Source: PLoS Genet. 2014 Nov 13;10(11):e1004764. doi: 10.1371/journal.pgen.1004764 (PMC4230838; doi:10.1371/journal.pgen.1004764)
Supplement: Table S3 — Sequences of primers used in the quantitative RT-PCR experiments. (DOCX) [file pgen.1004764.s013.docx]

| **Primer name** | **Primer sequence (5' to 3')** |
| --- | --- |
| *dilp2* F | GTATGGTGTGCGAGGAGTAT |
| *dilp2* R | TGAGTACACCCCCAAGATAG |
| *dilp3* F | AAGCTCTGTGTGTATGGCTT |
| *dilp3* R | AGCACAATATCTCAGCACCT |
| *dilp5* F | AGTTCTCCTGTTCCTGATCC |
| *dilp5* R | CAGTGAGTTCATGTGGTGAG |
| *erk7* F1 | GATTCCAAGCCAGGAACGTA |
| *erk7* R1 | TGTAGATGCGTATCGGAGGA |
| *erk7* F2 | GCGGAGATGGATCTTCACAT |
| *erk7* R2 | ATCTGGCTTGCTTCGTGACT |
| *inr* F | AACGAAAATGCTACGGATCG |
| *inr* R | GGGCACGCAAATAGGACTTA |
| *actin* F | CCGTACCACAGGTATCGTGTTG |
| *actin* R | GTCGGTTAAATCGCGACCG |
| *gapdh* F | CGAAGATCGGAATTAACGGA |
| *gapdh* R | ACCGTGAGTCGAGTCGAATT |
